# Evaluation of ^99m^Tc-3PRGD_2_ integrin receptor imaging in hepatocellular carcinoma tumour-bearing mice: comparison with ^18^F-FDG metabolic imaging

**DOI:** 10.1007/s12149-017-1173-4

**Published:** 2017-05-04

**Authors:** Jieling Zheng, Weibing Miao, Chao Huang, Haoxue Lin

**Affiliations:** Department of Nuclear Medicine, The First Affiliated Hospital of Fujian Medical University, 20 Chazhong Road, Taijiang District, Fuzhou, 350005 China

**Keywords:** ^99m^Tc-3PRGD_2_, Hepatocellular carcinoma, Integrin αvβ3, ^18^F-FDG

## Abstract

**Objective:**

Our study was designed to explore the utility of ^99m^Tc-HYNIC-PEG_4_-E[PEG_4_-c(RGDfK)]_2_ (^99m^Tc-3PRGD_2_) for the detection of hepatocellular carcinoma (HCC) and specifically to compare the diagnostic performance of ^99m^Tc-3PRGD_2_ integrin receptor imaging and 2-18-fluoro-2-deoxy-d-glucose (^18^F-FDG) metabolic imaging in a nude mouse model.

**Methods:**

^99m^Tc-3PRGD_2_ was synthesized using a HYNIC-3PRGD_2_ lyophilized kit with ^99m^TcO_4_ labelling. The nude mouse animal model was established by subcutaneously injecting 5 × 10^7^/ml HepG2 cells into the shoulder flank of each mouse. Biodistribution studies were performed at 0.5, 1, 2 and 4 h after intravenous administration of 0.37 MBq of ^99m^Tc-3PRGD_2_. Immunohistochemistry was performed to evaluate the expression level of integrin αvβ3 in the HCC tissues. Dynamic imaging was performed using list-mode after the administration of 55.5 MBq of ^99m^Tc-3PRGD_2_, to reconstruct the multiphase images and acquire the best initial scan time. At 8, 12, 16, 20 and 24 days after inoculation with HepG2 cells, 55.5 MBq of ^99m^Tc-3PRGD_2_ and 37 MBq of ^18^F-FDG were injected successively into the nude mouse model, subsequently, simultaneous SPECT/PET imaging was performed to calculate the tumour volume and tumour uptake of ^99m^Tc-3PRGD_2_ and ^18^F-FDG.

**Results:**

The biodistribution study first validated that the tumour uptake of ^99m^Tc-3PRGD_2_ at the different time points was higher than that of all the other organs tested in the experiment, except for the kidney. Integrin αvβ3 expressed highly in early stage HCC and declined for further necrosis of the tumour tissue. Subcutaneous tumours were visualized clearly with excellent contrast under ^99m^Tc-3PRGD_2_ SPECT/CT imaging, and the multiphase imaging comparison showed the tumours were prominent at 0.5 h, suggesting that the best initial scan time is 0.5 h post-injection. The comparison of the imaging results of the two methods showed that ^99m^Tc-3PRGD_2_ integrin receptor imaging was more sensitive than ^18^F-FDG metabolic imaging for the detection of early stage HCC, meanwhile the tumour uptake of ^99m^Tc-3PRGD_2_ was consistently higher than that of ^18^F-FDG. However, as tumour necrosis further increased in HCC tissues, the uptake of ^18^F-FDG was higher than that of ^99m^Tc-3PRGD_2_.

**Conclusion:**

Our study demonstrated that ^99m^Tc-3PRGD_2_ is a valuable tumour molecular probe for the detection of early stage HCC compared with ^18^F-FDG, meriting further investigation of ^99m^Tc-3PRGD_2_ as a novel SPECT tracer for tumour imaging.

## Introduction

Hepatocellular carcinoma (HCC) is a primary cancer of the liver with a significant impact worldwide due to its high incidence and mortality. Early detection and diagnosis of HCC can bring great benefit for the prognosis of patients. Currently, the definitive diagnosis of HCC depends primarily on conventional imaging modalities, such as ultrasonography, magnetic resonance imaging or computed tomography [1]. However, these techniques are usually based on anatomical, morphological or haemodynamic alterations. The appearance of HCC at the early stage is atypical, and thus, there is no obvious difference between small HCC and preneoplastic nodules. Moreover, the types of hepatic lesions are complicated, various forms of inflammation, granulomatous lesions and benign tumours can mimic the morphology and haemodynamics of early HCC, resulting in the difficult differential diagnosis of early HCC in non-cirrhotic patients. Therefore, conventional imaging modalities still have some limitation in the diagnosis of early HCC.

Hepatocarcinogenesis is considered a multi-step process; changes firstly come from gene, molecule and metabolic function, with subsequent anatomical and morphological changes. Hence, acquiring cellular, molecular or functional information on HCC tumours through functional imaging techniques has significant clinical value for the early detection and diagnosis of HCC. Nowadays, 2-18-fluoro-2-deoxy-d-glucose (^18^F-FDG) PET/CT is the most widely employed tumour-functional imaging modality worldwide, however, the positive detection rate for HCC is still not ideal, particularly in terms of the low specificity and sensitivity for the detection of early stage HCC [2, 3]. Thus, a large number of other compounds have been investigated as an alternative or complement to ^18^F-FDG, most recently ^11^C-acetate [4, 5], ^11^C-choline [6], and ^18^F-labelled fluorothymidine [7, 8]; however, none of these compounds have gained broad approval. Accordingly, there is still a distinct need for better functional imaging modalities available for patients with HCC.

Integrin αvβ3, a member of the integrin receptor family, is highly expressed in both tumour cells and activated endothelial cells and plays a pivotal role in tumour growth, invasion and metastasis [9–11]. The tri-peptide sequence of arginine-glycine-aspartic acid (RGD) is a cellular recognition site that can selectively bind to integrin αvβ3 [12]. A variety of radiolabeled RGDs have been developed as SPECT and PET radiotracers for tumour imaging and have attracted considerable attention in recent decades. Our study was designed to investigate the feasibility of ^99m^Tc-HYNIC-PEG_4_-E[PEG_4_-c(RGDfK)]_2_ (^99m^Tc-3PRGD_2_) for the detection of HCC and specifically to compare the diagnostic performance of ^99m^Tc-3PRGD_2_ integrin receptor imaging and ^18^F-FDG metabolic imaging in HCC tumour-bearing mice.

## Materials and methods

### Radiopharmaceutical preparation

The HYNIC-3PRGD_2_ lyophilized kit was obtained from the Medical Isotope Research Center of Peking University. The synthesis of the labelling precursor and subsequent ^99m^Tc-labelling were performed according to previously described methods [13]. The vial containing the above product was heated at 100 °C for 20 min and then incubated at room temperature for 10 min. The resulting solution was analysed using radioactive thin layer chromatography to verify that the radiochemical purity was >95%. ^18^F-FDG was supplied by the Atom High-tech Company of China (Fuzhou, Fujian, China). Doses were prepared by dissolving the radiotracer in saline.

### Animal model

This study was approved by our hospital’s Ethics Committee for medical research. A total of 36 female athymic nu/nu mice were purchased from the Medical Animal Center, FuZhou General Hospital of Nanjing Military Command (6-week-old, average body weight 24.16 ± 2.28 g). The human HCC HepG2 cell line was purchased from the Institute of Cytobiology, Chinese Academy of Science. The HepG2 cells were cultured in DMEM supplemented with 10% foetal bovine serum (FBS, HyClone) and 1% penicillin and streptomycin solution (Gibco) at 37 °C in a humidified atmosphere of 5% CO_2_ in air. The cells were split or harvested when they reached 90–100% confluency to maintain exponential growth. Each mouse was implanted subcutaneously with 5 × 10^7^/ml HepG2 cells into the left shoulder flank. The tumour volume was measured every other day with vernier callipers and calculated using the following formula: (length × width^2^)/2.

### Biodistribution study

A total of 16 HepG2 hepatoma-bearing mice, with a maximum tumour diameter of 1.0 cm, were randomly divided into 4 groups, with 4 animals in each group. Each mouse was injected intravenously with 0.37 MBq of ^99m^Tc-3PRGD_2_ in 0.1 ml. At 0.5, 1, 2 and 4 h after administration, the mice were sacrificed by humane euthanasia, and the important organs (tumour, heart, liver, spleen, lungs, kidneys, stomach, intestine, muscle and blood) were excised, dried, weighed and counted using a γ-counter. The percentage of the injected dose per gram (%ID/g) and the target/non-target (*T*/NT) ratios were calculated to evaluate the biological distribution characteristics of ^99m^Tc-3PRGD_2_ in the nude mice.

### Immunohistochemistry

In total, 12 mice were chosen randomly for HepG2 cell inoculation at different time points. When the subcutaneous tumours grew to various sizes (0.06–1.0 g), the mice were sacrificed by humane euthanasia, and the tumours were excised, fixed in 10% formalin, embedded in paraffin, cut into slices (6-μm-thick) and dried. After dewaxing in xylene and hydrating in gradient ethyl alcohol (from 100 to 80%), the sections were incubated with the mouse anti-integrin αvβ3 monoclonal antibody (1:100, Abcam, Cambridge, USA), followed by sequential incubation with reagent A (reaction enhancer) and B (enzyme-conjugated goat anti-mouse/rabbit IgG polymer) of an immunohistochemical EliVision plus kit, then washed with phosphate buffer solution (PBS) and stained with fresh diaminobenzidine (DAB) solution. Finally, the sections were visualized under an Olympus optical microscope (Olympus Corporation, Tokyo, Japan). The appearance of brown colouring in the cytoplasm or cytomembrane of the tumour tissues was regarded as positive integrin αvβ3 expression.

### Dynamic SPECT/CT imaging with ^99m^Tc-3PRGD_2_

All imaging studies were performed using microSPECT/PET/CT equipment (Versatile Emission Computed Tomography System, Milabs, Utrecht, the Netherlands), which enables simultaneous submillimeter imaging of single-photon and positron-emitting radiolabeled molecules. Four HepG2 hepatoma-bearing mice were injected with 55.5 MBq of ^99m^Tc-3PRGD_2_ in 0.1 ml saline via the tail vein. At 20 min after administration, the mice were quickly transferred into a shielded chamber connected to an isoflurane anaesthesia unit. After the induction of anaesthesia, the mice were immediately placed supine on the scanning bed. The ^99m^Tc-3PRGD_2_ imaging commenced 30 min after the tracer injection. First, rectangular scans in the regions of interest (ROIs) from both the SPECT and CT were selected by X-ray. CT scanning was performed using the “normal” acquisition settings at 55 keV and 615 μA, and then SPECT data were acquired from 30 min to 3 h and 4 to 4.5 h post-injection (pi) in list-mode using the following settings: 84 projections per frame over 30 min and a 20% energy window centred on 140 keV.

### Multiphase image reconstruction and data analysis

CT reconstruction was performed using a cone-beam filtered back-projection algorithm (NRecon v1.6.3, Skyscan, Belgium). SPECT image reconstruction of the list-mode raw data was performed using a pixel-based ordered-subsets expectation maximization algorithm (POSEM), with the initial scan time set as 0.5, 1, 2 and 4 h pi (a 30-min scanning time). The new SPECT data and CT data were fused together using PMOD software (PMOD Technologies, Zurich, Switzerland). To achieve a higher signal-to-noise ratio, Gaussian smooth 3D (1.0 mm full-width at half maximum) was used and the image colour gradation was adjusted. For each SPECT/CT fusion image, the ROI covering the entire tumour was generated automatically by setting a maximum pixel value threshold of 30% or more, and then the tumour volume and radioactivity counts were calculated using PMOD. The tumour uptake of ^99m^Tc-3PRGD_2_ was expressed as the percentage of the injected dose (%ID) and the percentage of the injected dose per unit volume (%ID/cm^3^). The background ROI (0.01 cm^3^) was drawn over the muscle of the contralateral forelimb, and then the *T*/NT ratio of tumour to muscle was calculated for the semi-quantitative analysis.

### Simultaneous SPECT/PET Imaging with the ^99m^Tc/^18^F dual-isotope

The HepG2 hepatoma-bearing mice were reared at 26–27 °C. Four mice were chosen for simultaneous SPECT/PET imaging at 8, 12, 16, 20 and 24 days after inoculation with HepG2 cells, and they were fasted but allowed to drink water for 12 h before imaging. The selected mice were injected intravenously with 55.5 MBq of ^99m^Tc-3PRGD_2_ and 37 MBq of ^18^F-FDG successively. A 45-min simultaneous SPECT/PET acquisition was obtained, beginning at 45 min after the tracer injection. Throughout the scanning, the body temperature of the mice was monitored using a constant heating and feedback device. To achieve signal separation of the two tracers, the energy peak was set to 140 keV for ^99m^Tc-3PRGD_2_ and 511 keV for ^18^F-FDG. All photopeak windows were set to a width of 20%. The SPECT and PET images were reconstructed using POSEM. The tumour volume and tumour uptakes of ^99m^Tc-3PRGD_2_ and ^18^F-FDG (%ID and %ID/cm^3^) were calculated using PMOD software.

### Statistical analyses

The quantitative data are expressed as $\bar{x}\; \pm \;s$. The statistical analyses were performed using one-way analysis of variance (ANOVA) for multiple comparisons, least significant difference (LSD) *t* test for the comparisons between groups and regression analyses for the correlation analysis with the SPSS 18.0 statistical software package. The level of significance was set at *P* < 0.05.

## Results

### Biodistribution of ^99m^Tc-3PRGD_2_

**Fig. 1:**
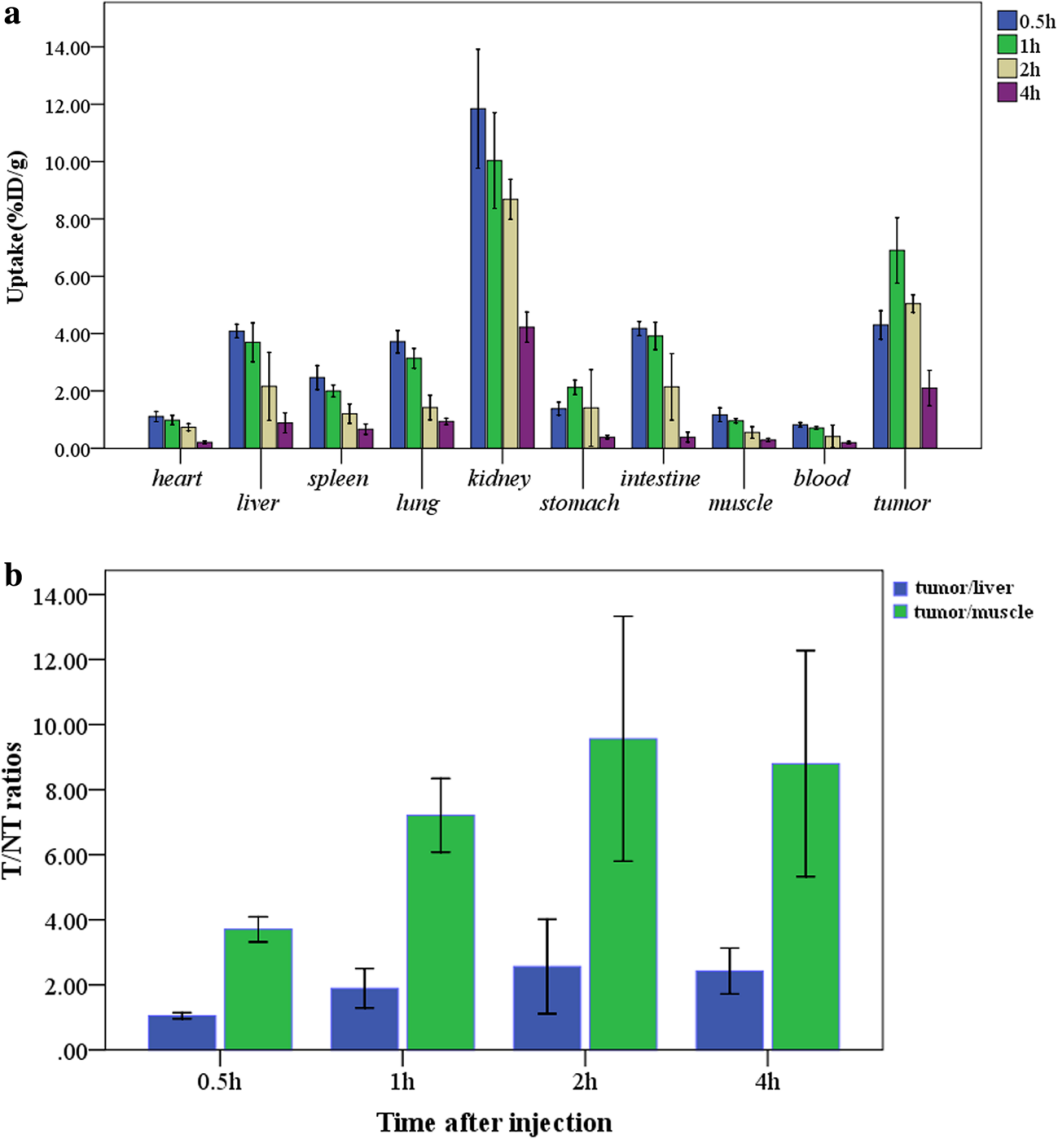
**a** The biodistribution data in selected organs of HepG2 hepatoma-bearing mice obtained at 0.5, 1, 2 and 4 h after injection of 0.37 MBq of ^99m^Tc-3PRGD_2_ (*x* ± *s*, *n* = 4). **b** Comparison of the *T*/NT ratios of tumour to muscle and tumour to liver in HepG2 hepatoma-bearing mice at 0.5, 1, 2 and 4 h after injection of 0.37 MBq of ^99m^Tc-3PRGD_2_ (*x* ± *s*, *n* = 4)

As shown in Fig. 1a, the tumour uptake of ^99m^Tc-3PRGD_2_ at the different time points was 4.301 ± 0.313 %ID/g (0.5 h), 6.902 ± 0.717 %ID/g (1 h), 5.045 ± 0.193 %ID/g (2 h) and 2.099 ± 0.388 % ID/g (4 h), with a statistically significant difference between each group (*F* = 78.864,*P* < 0.05). ^99m^Tc-3PRGD_2_ was primarily excreted through the urinary system as evidenced by the higher renal uptake (11.842 ± 1.304, 10.034 ± 1.051, 8.683 ± 0.437, 4.225 ± 0.332 %ID/g at 0.5, 1, 2 and 4 h pi, respectively). Moderate accumulation was also found in the intestine and liver, illustrating that ^99m^Tc-3PRGD_2_ was partially metabolized by the liver or intestine. Other organs with relatively low radioactivity were the spleen, lung, stomach, blood and muscle. Figure 1b shows the *T*/NT ratios of tumour to muscle and tumour to liver, indicating that the highest *T*/NT ratios were obtained at 2 h pi (9.57 ± 2.37 and 2.56 ± 0.91, respectively).

### Integrin αvβ3 expression in HCC tissues

**Fig. 2:**
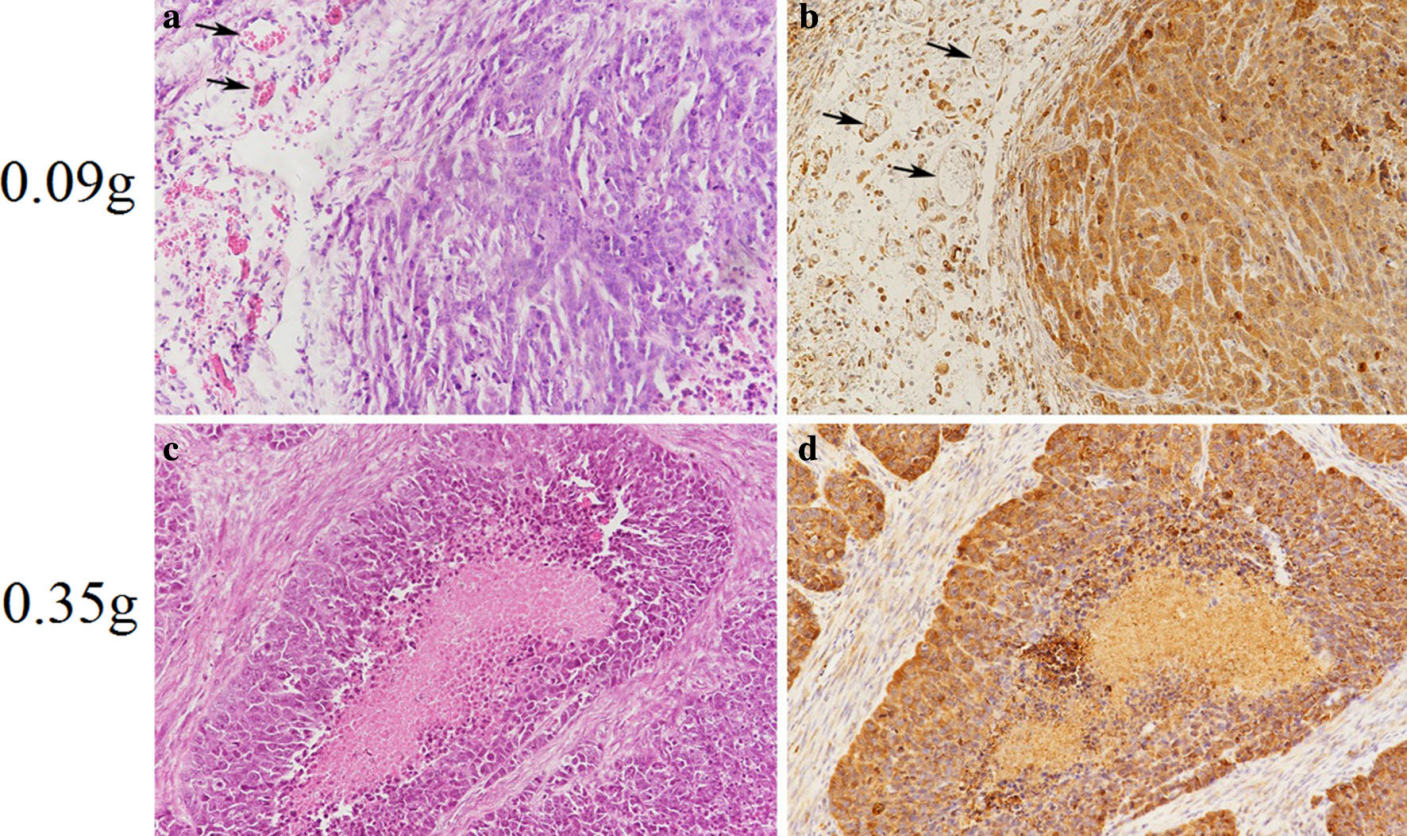
Representative microscopic images of the tumour slices from the different sized xenografted HepG2 tumours after haematoxylin-eosin (HE) staining and immunohistochemical staining (0.09 and 0.35 g). **a** HE staining showed the HCC cells and tumour blood vessels without tumour necrosis. **b** Immunohistochemistry showing the positive expression of integrin αvβ3 was visualized as brown staining. The results showed that αvβ3 was predominantly expressed in the cytoplasm and cytomembrane, as well as the vascular epithelial cells (*arrows*). **c** HE staining showing the central visual field was characterized by cell necrosis. **d** Immunohistochemistry showing that αvβ3 staining was negative in the necrotic region. The images were obtained by an Olympus optical microscope (10 × 20 *magnification*)

HCC cells have large and atypical nuclei and mitosis in haematoxylin-eosin (HE)-stained slices. Immunohistochemistry showed that integrin αvβ3 positive expression, as visualized by brown staining, was observed predominantly in the cytoplasm and cytomembrane, as well as the vascular epithelial cells (Fig. 2). The expression level of αvβ3 in HCC cells varied among the different tumour sizes (0.06–1.0 g). When the tumour weight was less than 0.23 g without tumour necrosis, the overall expression level of αvβ3 was relatively high. When the tumour weight was between 0.35 and 1.0 g, the expression level of integrin αvβ3 gradually declined in line with the necrosis of the HCC tissues.

### Dynamic SPECT/CT imaging results

**Fig. 3:**
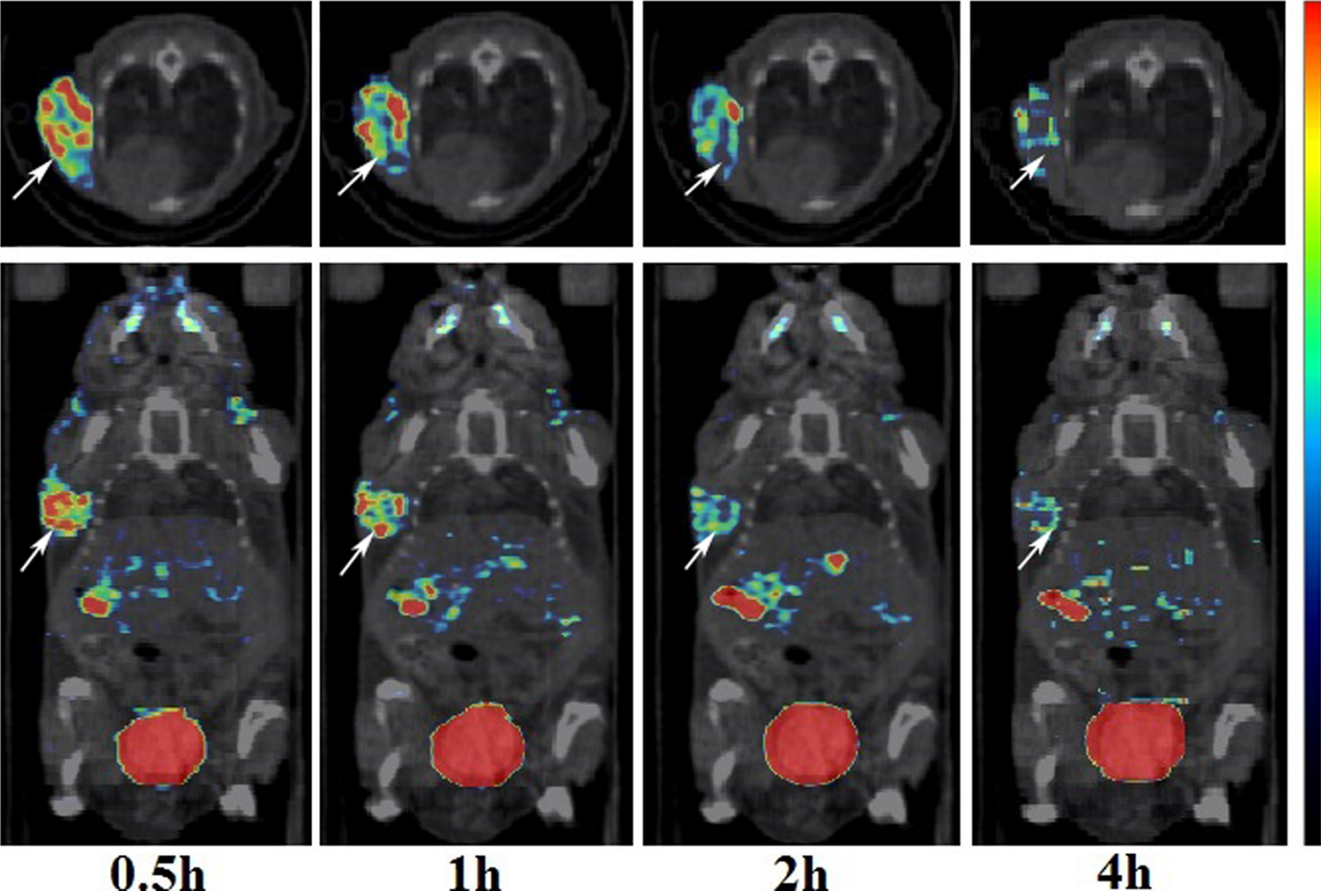
The *traverse views* (**a**) and *coronal views* (**b**) of multiphase SPECT/CT images after the injection of 55.5 MBq of ^99m^Tc-3PRGD_2_ in HepG2 hepatoma-bearing mice, with the initial scan time set as 0.5, 1, 2 and 4 h pi (a 30-min scanning time)

The subcutaneous tumours were clearly visualized under multiphase SPECT/CT imaging, with excellent contrast at 0.5, 1, 2 and 4 h pi (Fig. 3). The accumulation of radioactivity in the subcutaneous tumours was most obvious when the initial scan time was 0.5 h pi, and some radionuclide remained until 4 h pi. Prominent renal uptake of ^99m^Tc-3PRGD_2_ was observed at all time points examined, whereas abdomen uptake was moderate and radioactivity accumulation in the lung, mediastinum and muscle was relatively low. Furthermore, radioactivity distribution in all body tissue types decreased over time. These results were consistent with that of the biodistribution study.

The tumour uptake was 4.61 ± 1.17 %ID/cm^3^ (0.5 h), 3.61 ± 0.87 %ID/cm^3^ (1 h), 3.02 ± 0.75 %ID/cm^3^ (2 h) and 2.63 ± 0.71 %ID/cm^3^ (4 h), suggesting that the highest tumour uptake occurred when the initial scan time was 0.5 h pi. The *T*/NT ratios of tumour to muscle were 3.39 ± 1.33 (0.5 h), 3.17 ± 1.33 (1 h), 3.66 ± 0.92 (2 h) and 2.86 ± 0.49 (4 h). Although the highest *T*/NT ratio was found when the initial scan time was 2 h pi, there were no statistically significant differences among the four groups (*P* > 0.05). Both the direct visualization and quantitative analysis indicated that the best initial scan time was 0.5 h pi.

### Multimodal imaging of HCC with the ^99m^Tc/^18^F dual-isotope

**Fig. 4:**
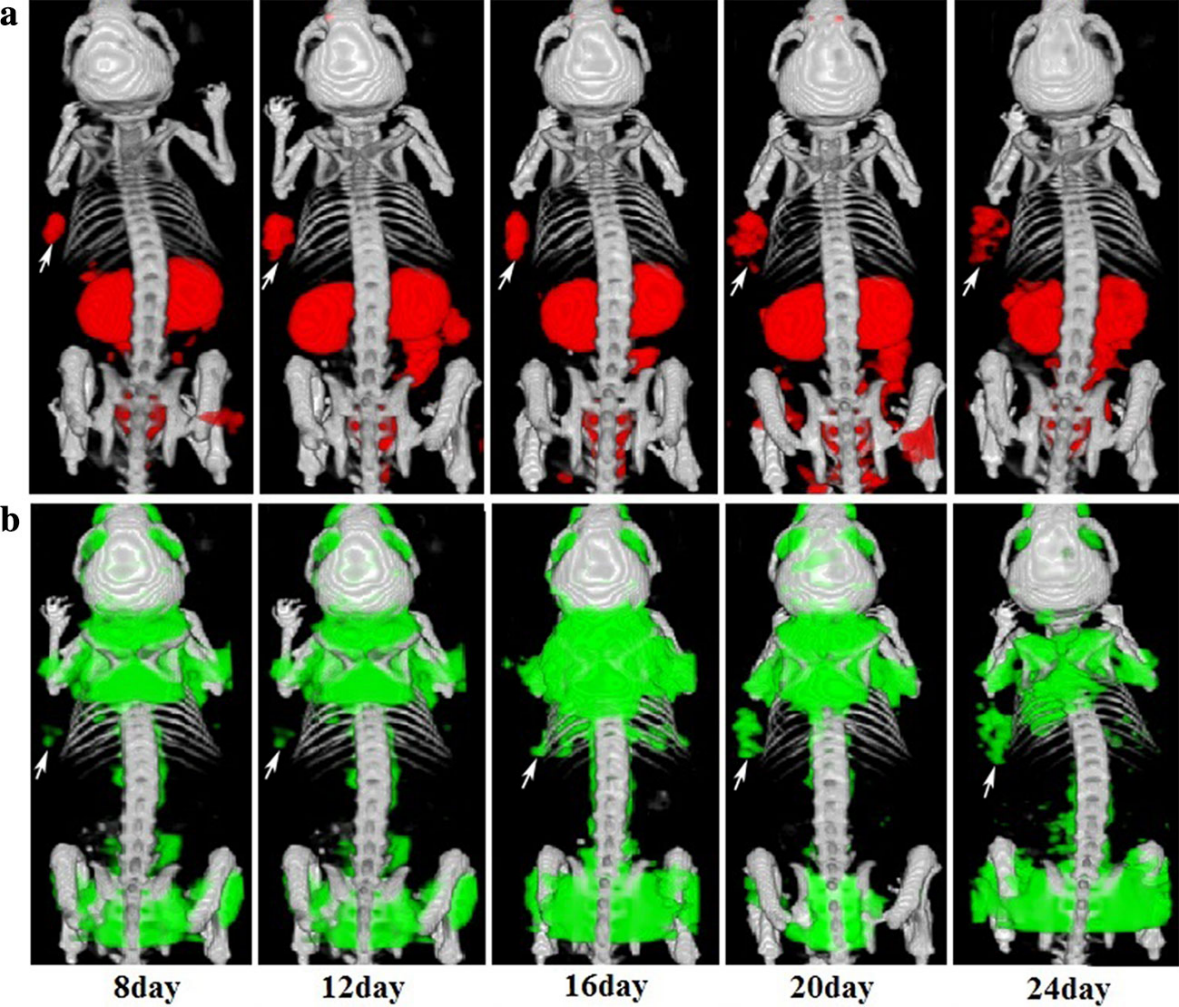
The three-dimensional views of simultaneous SPECT/PET images obtained 45 min after the injection of 55.5 MBq of ^99m^Tc-3PRGD_2_ and 37 MBq of ^18^F-FDG in ^99m^Tc-window (**a**) and ^18^F-widow (**b**), with a 45-min scanning time. These images were acquired at 8, 12, 16, 20 and 24 days after HepG2 cell inoculation

We assessed the performance differences between ^99m^Tc-3PRGD_2_ integrin receptor imaging and ^18^F-FDG metabolic imaging during the growth of tumour with the microSPECT/PET/CT system (Fig. 4). ^99m^Tc-3PRGD_2_ SPECT/CT could sensitively detect subcutaneous tumours at 8 days after inoculation with HepG2 cells. The tumours were clearly visualized at 8, 12, and 16 days after inoculation with HepG2 cells for the distinct concentration and homogenous distribution of radionuclides, and the tumour volume gradually increased over time. In contrast, there was only minimal uptake in the subcutaneous tumours under ^18^F-FDG PET/CT imaging at 8, 12, and 16 days after inoculation with HepG2 cells. From day 20 to day 24, as tumour necrosis further increased, both the ^99m^Tc-3PRGD_2_ SPECT/CT fusion images and ^18^F-FDG PET/CT fusion images showed uneven tracer distribution in the HCC tissues. Notably, the uptake of ^18^F-FDG into the brown adipose tissue located in the neck and back or multiple muscle tissues was too high, thereby affecting image quality and forcing some measures including warming, fasting and anaesthesia to be performed in advance.

### Comparison of tumour uptake of ^99m^Tc-3PRGD_2_ and ^18^F-FDG

**Fig. 5:**
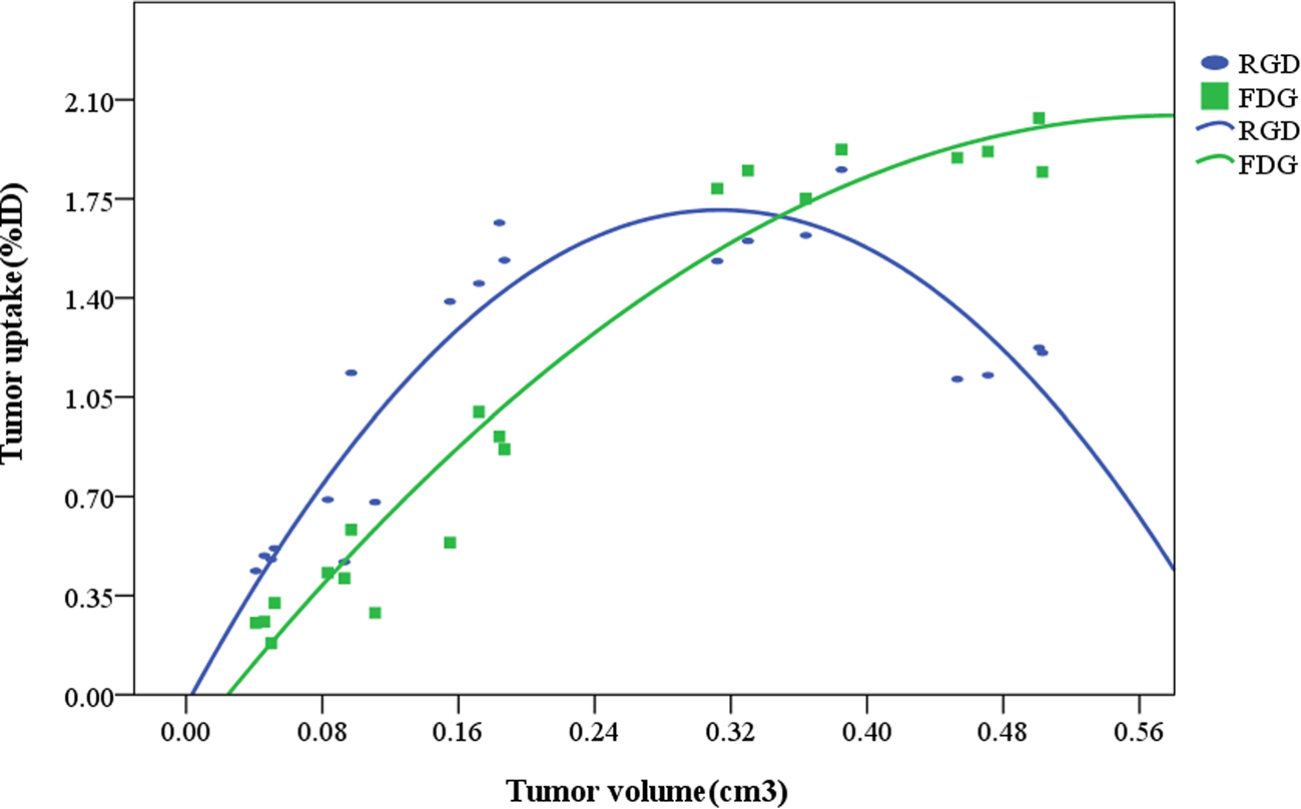
The *blue line* represents the relationship between the tumour volume (cm^3^) and tumour uptake of ^99m^Tc-3PRGD_2_ (%ID) at 8, 12, 16, 20 and 24 days after HepG2 cell inoculation. The *green line* represents the relationship between the tumour volume (cm^3^) and tumour uptake of ^18^F-FDG (%ID) at 8, 12, 16, 20 and 24 days after HepG2 cell inoculation

The tumour uptake of ^99m^Tc-3PRGD_2_ and ^18^F-FDG at the different imaging time points is shown in Table 1. Except for the uptake measurements on day 20 (*P* *<* 0.05), there were no statistically significant differences among the different groups, which were divided according to the imaging day (*t* = 1.62, *P* < 0.05). A regression analysis was used to assess the relationship between the tumour volume and tumour uptake of ^99m^Tc-3PRGD_2_ and ^18^F-FDG (Fig. 5). The relationship between the tumour volume and %ID of ^99m^Tc-3PRGD_2_ was modelled as quadratic polynomial, with an *R* ^2^ value of 0.85. The conversion formula was *y* = −17.81*x* ^2^ + 11.16*x*−0.38, indicating that the uptake of ^99m^Tc-3PRGD_2_ increased to its peak as the tumour volume increased and then declined due to necrosis (day 24). The relationship between the tumour volume and %ID of ^18^F-FDG was also modelled as quadratic polynomial, with an *R* ^2^ value of 0.96. The conversion formula was *y* = −6.61*x* ^2^ + 7.68*x*−0.18, illustrating that the tumour uptake of ^18^F-FDG was consistently less than that of ^99m^Tc-3PRGD_2_ from day 8 to day 16. When all the tumour volume was greater than 0.30 cm^3^ from day 20 to day 24, the uptake of ^18^F-FDG, in turn, was higher than that of ^99m^Tc-3PRGD_2_ during this time period.

**Table 1:** Comparison of tumor uptakes of ^99m^Tc-3PRGD_2_ and ^18^F-FDG at 8, 12, 16, 20 and 24 days after inoculation with HepG2 cells (%ID, $\bar{x}\; \pm \;s$, *n* = 4)

Tracer	8 days	12 days	16 days	20 days	24 days	*F*	*P*
^99m^Tc-3PRGD_2_	0.48 ± 0.03	0.79 ± 0.23	1.51 ± 0.12	1.65 ± 0.14	1.17 ± 0.06	*F* = 52.73	*P* < 0.05
^18^F-FDG	0.25 ± 0.06	0.43 ± 0.12	1.04 ± 0.14	1.80 ± 0.12	1.88 ± 0.15	*F* = 153.20	*P* < 0.05

## Discussion

Among the various forms of radioactive medications, ^99m^Tc-3PRGD_2_ stands out for its superior αvβ3-targeting ability, biodistribution and pharmacokinetics [13–15]. It has been shown to be a promising tracer for the diagnosis, staging, and treatment evaluation of thoracic malignancies such as lung cancer, breast cancer and metastasis [16–19]. However, few studies have investigated the use of ^99m^Tc-3PRGD_2_ for the diagnosis of HCC, especially no studies have compared the efficacy of ^99m^Tc-3PRGD_2_ integrin receptor imaging with ^18^F-FDG metabolic imaging. Integrin αvβ3 express lowly in normal liver tissues including hepatocytes, stellate cells, Kupffer cells, and other nonparenchymal cells [20]. Studies have reported that the expression of integrin αvβ3 is significantly higher in HCC tissues than in adjacent normal hepatic tissue [21]. In this study, we demonstrated that HCC could be detected using SPECT/CT with ^99m^Tc-3PRGD_2_ as the radiotracer.

To further estimate the usefulness of ^99m^Tc-3PRGD_2_ in the non-invasive monitoring of HCC tumours, ^18^F-FDG was chosen as the positive control. The imaging results indicated that ^99m^Tc-3PRGD_2_ SPECT/CT has an advantage over ^18^F-FDG PET/CT for HCC imaging in two aspects. First, ^99m^Tc-3PRGD_2_ SPECT/CT could distinctly and sensitively detect the HCC tissues at 8 days after HepG2 cell inoculation, a time point at which the smallest tumour volume was only 0.041 cm^3^ but the uptake of ^99m^Tc-3PRGD_2_ reached 10.67 %ID/cm^3^ at this point. In contrast, the tumours were unclear under ^18^F-FDG images so the imaging could not be used to accurately detect early stage HCC. Furthermore, the tumour uptake of ^99m^Tc-3PRGD_2_ was significantly higher than that of ^18^F-FDG from day 8 to day 16. This finding suggests that ^99m^Tc-3PRGD_2_ has the potential to be used as a SPECT tracer for early detection of HCC over ^18^F-FDG. Second, it is well known that ^18^F-FDG PET/CT imaging is affected by many factors, such as glucose levels, insulin, and temperature. Muscle or brown adipose tissue can also competitively uptake ^18^F-FDG [22, 23]. In contrast, ^99m^Tc-3PRGD_2_ integrin receptor imaging shows less interference from these factors, resulting in the acquisition of clearer images. Moreover, ^99m^Tc-3PRGD_2_ can be more widely applied to improve the screening rates for early HCC detection because it is more cost-effective, feasible and practical as a SPECT tracer.

We analysed the possible reasons for the different imaging results with ^99m^Tc-3PRGD_2_ and ^18^F-FDG comes from two completely different biological processes they are targeting. In this study, we found that αvβ3 expression in the xenografted HepG2 tumours was high in the early stage and showed an increasing trend until the tumours became necrotic. Given that ^99m^Tc-3PRGD_2_ is a target-specific radiotracer whose biodistribution is determined by its receptor, the high expression level of αvβ3 in early HCC contributes to the high tumour uptake of ^99m^Tc-3PRGD_2_; therefore, ^99m^Tc-3PRGD_2_ imaging is able to sensitively detect early HCC while ^18^F-FDG imaging is used to evaluate glucose metabolism, the main mechanism of ^18^F-FDG uptake in malignant tumours largely depends on the presence of facilitated glucose transporters, especially type 1 (Glut 1) and a rate-limiting glycolytic enzyme, especially hexokinase (HK) type II. However, HCCs have different glucose-regulating mechanisms and enzyme expression patterns, and the low expression of Glut 1 and the existence of dephosphorylase are the main reasons for the low ^18^F-FDG uptake [24–26]. The uptake of ^18^F-FDG in HCC is also related to the degree of differentiation and lesion size, with well-differentiated HCC exhibiting a high rate of gluconeogenesis compared with that in normal liver tissues, resulting in similar uptake of ^18^F-FDG [27]. In particular, early stage HCC tumours, which are well differentiated and small in size, show low radioactivity concentrations under ^18^F-FDG PET/CT imaging.

Except ^99m^Tc-3PRGD_2_ SPECT/CT was more sensitive than ^18^F-FDG PET/CT for the detection of early HCC, it is worth noting that HCC uptake of ^18^F-FDG was higher than that of ^99m^Tc-3PRGD_2_ in advanced stage HCC, indicating that the diagnostic efficiency of ^18^F-FDG PET/CT might be better than ^99m^Tc-3PRGD_2_ SPECT/CT for imaging advanced HCC. On one hand, the tumour uptake of ^99m^Tc-3PRGD_2_ may have decreased due to the concomitant maturity of blood vessels, tumour necrosis, and larger interstitial space that accompanied the rapid growth of the tumour [28]. On the other hand, cancer cell growth is heavily dependent on glucose metabolism as a major energy substrate, particularly poorly differentiated advanced HCC. The association of neoplastic growth with increased aerobic glycolysis is known as the Warburg effect, which might help explain why the uptake of ^18^F-FDG increased in advanced stage HCC [29]. Therefore, combining ^99m^Tc-3PRGD_2_ SPECT/CT and ^18^F-FDG PET/CT imaging could aide in the detection of HCC by compensating for the deficiency of ^18^F-FDG in the detection of early stage HCC and ^99m^Tc-3PRGD_2_ in the diagnosis of advanced HCC. Moreover, multimodality imaging using multiple tracers can also provide complementary information on tumour-associated functions with excellent sensitivity and high selectivity; thus, multimodality imaging might improve the tumour diagnosis, differential diagnosis and therapeutic monitoring of HCC compared with those of a single radiological modality alone.

In the complex pathogenesis of hepatic carcinoma, angiogenesis is one of the crucial events, relying on the migration and invasion of vascular endothelial cells, which is regulated by various cell adhesion receptors including the integrin family [30]. Among the 24 members of integrin family, αvβ3 is the most widely studied because its expression is an important factor in determining the invasiveness and metastatic potential of malignancy [11]. In our study, we demonstrated that αvβ3 was highly expressed on the activated endothelial cells of HCC tissues. Currently, sorafenib chemotherapy, an anti-angiogenic therapy, is the first line therapy for HCC patients who are not eligible for surgical removal or liver transplantation. However, there is still no appropriate imaging modality to monitor the efficacy of anti-angiogenic therapy. Research has shown that ^99m^Tc-3PRGD_2_ can be applied to guide anti-angiogenic therapy by visualizing and quantifying the expression level of αvβ3 [18, 31]. Thus, ^99m^Tc-3PRGD_2_ imaging has the potential to select suitable patients who could benefit from sorafenib chemotherapy and supply an objective basis for its curative effect.

It should be noted that our results are preliminary and obtained using a very limited number of animals; therefore, insufficient data may have caused errors in our analysis. Further studies with a substantial number of animals or a comparable translational human clinical trial are still needed. Moreover, most HCCs arise in the setting of chronic hepatitis induced by HCV or HBV infection. However, current animal models cannot mimic the inflammatory microenvironment that leads to the pathogenesis of HCC and the pathogenic sequence of human HCC that starts with fibrosis and cirrhosis prior to the development of HCC. Several studies have reported that ^99m^Tc-3PRGD_2_ can assess activation of hepatic stellate cells and diagnose liver fibrosis [32, 33]. The uptake in normal hepatic, hepatitis and liver fibrosis tissues may decrease the *T*/NT ratio and influence the detection of HCC. Finally, the relation between the pathologic grade and tumour uptake of ^99m^Tc-3PRGD_2_ was not assessed in the present study; therefore, we cannot verify whether various pathologic types of liver cancer impact ^99m^Tc-3PRGD_2_ SPECT/CT imaging.

In conclusion, ^99m^Tc-3PRGD_2_ is more sensitive for the detection of early stage HCC than ^18^F-FDG, but in advanced HCC, the diagnostic performance of ^18^F-FDG might be better than that of ^99m^Tc-3PRGD_2_ because the tumour uptake of ^99m^Tc-3PRGD_2_ significantly declined due to tumour necrosis. Combined ^99m^Tc-3PRGD_2_ SPECT/CT and ^18^F-FDG PET/CT imaging would be helpful for the imaging diagnosis of HCC. We believe that ^99m^Tc-3PRGD_2_ is worth further investing as a promising radiotracer for the diagnosis of HCC and guidance of the anti-angiogenic therapy.
